# Demonstrating completeness in optical neural computing

**DOI:** 10.1038/s41377-025-02123-2

**Published:** 2026-01-03

**Authors:** Krzysztof Tyszka

**Affiliations:** Institute of Experimental Physics, Faculty of Physics, University of Warsaw, ul. Pasteura 5, Warsaw, PL-02-093 Poland

**Keywords:** Integrated optics, Optoelectronic devices and components

## Abstract

A silicon photonic deep optical neural network integrating convolutional and fully connected layers with on-chip optoelectronic nonlinear activations operates with partially coherent light to achieve high-speed, energy-efficient, end-to-end inference. This demonstration establishes a functional and scalable platform for evaluating complete optical neural processing, representing another step toward specialised, ultrafast photonic architectures beyond electronics.

Artificial Neural Networks (ANNs) are trainable algorithms inspired by the structure and functioning of the brain, designed for solving classification or regression tasks by training on extended datasets. These algorithms lie at the core of today’s progress in the AI field, being an essential part of large language models and transformer architecture as one of the most prominent examples. Although introduced in the 1940s by McCulloch and Pitts^1^, only in the last three decades has it gained increased attention because of the error back-propagation algorithm proposed by Rumelhart, Hinton, and Williams^2^. This enabled the training of multi-layer networks and effectively led to the demonstration of today’s large and deep models with billions of parameters. But this technological step could not have been done without the parallel development of digital electronic devices capable of more and more efficient computations, including CPUs, GPUs, and, more recently, processing units solely devoted to the acceleration of ANN computations. The most recent progress also sparked interest in other fields, motivated by the collapse of electronic scaling “laws”, as further development of AI inevitably relies on and demands high computational efficiency^3^.

Particularly, this led to the resurrection of the analog computing paradigm - abandoned in the past because of the unmatched universality of digital computing - based on the assumption that analog computing enables asynchronous, highly parallel processing and in-memory computing, hardware-emulating the inherent properties of ANN algorithms. A similar belief is at the root of the reborn of the optical analog computing field, although with the additional motivation of capitalizing on the unmatched high throughput of optical links inherited from telecom technology^4^.

This comeback of optical computing over the past decade was also strongly motivated by the maturation of silicon integrated photonics technology, which coincided with the rapid rise of deep learning algorithms relying heavily on matrix multiplication operations repeated across many layers, often billions of times during training. Because matrix multiplication dominates both computation time and energy use, optimizing this operation became central to accelerating AI computations (as shown in a simplified scheme, Fig. 1a). As a result, optical approaches have been viewed as a promising route to handle the large-scale linear algebra, seeing rapid development from proof of principle demonstration in 2017 by Shen et al. towards recently demonstrated complete hybrid accelerators with photonic tensor cores^5–7^. This successful route stemmed from the hybrid approach, where photonics were used for the acceleration of specific operations, while a configurable electronic interface provided universality and usefulness of the device.

**Fig. 1:**
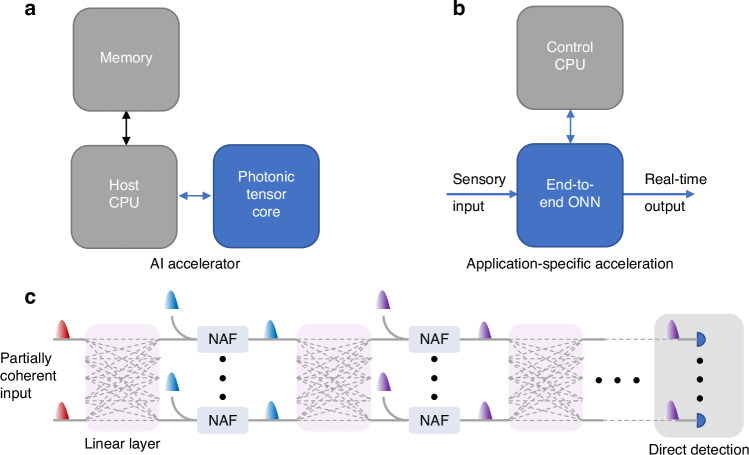
Two use cases of photonic acceleration. **a** Acceleration of linear algebra operations using a photonic tensor core, where network layer outputs are sequentially computed and the CPU offloads matrix operations to the photonic hardware. **b** An interfaced end-to-end optical neural network that accelerates inference tasks in sensory applications, enabling high-speed, real-time processing. **c** Simplified schematic of an end-to-end optical neural network architecture from ref. ^24^, comprising matrix multiplication layers, optically driven nonlinear activators (NAF), and operating with partially coherent light

Meanwhile, the number of publications describing theoretically or demonstrating functional components and architectures of end-to-end Optical Neural Networks (ONN) has rapidly increased^8^. As for now, the burden of analog technology still prevents demonstrations of optical deep networks, which can compete with the scalability of electronics, with the main reason being footprint constraints and cascadability issues^9,10^. Although large-scale deep networks are still ahead, the high-speed processing capabilities of such hardware can be harnessed for practical solutions based on shallower networks in specific domains, especially those requiring real-time sensing and processing at GHz rates^11^. In such, as schematically shown in Fig. 1b, optical solutions have the potential to outperform electronics already now, taking advantage of non-von-Neumann architectures^12^ and lightspeed transfers^13–15^. However, shifting from basic research towards application-specific designs requires demonstrations of complete end-to-end networks that can be treated as a test-bed for verification of future use. Here, the hindering is related to the lack of configurable energy-efficient components, realizing rapidly switching non-linear activation function (NAF) of optical neurons^14,16,17^ - one of the critical neural network functionalities – to be combined with already demonstrated optical tensor core technologies^18^. Another challenges are related to material incompatibility, negative net gain, or lack of configurability in the case of optical NAFs, or relatively high complexity and lagging in optoelectronic NAFs, with both solutions often decreasing energy efficiency.

Despite these difficulties, a number of end-to-end photonic networks have already been demonstrated, ranging from free-space planar architectures to on-chip integrated systems employing various types of NAFs^19–23^. Building on these advances, a recent paper published in *Light: Science & Applications* takes a significant step forward by addressing key issues in achieving improved end-to-end on-chip photonic demonstrations.

Wu et al. report the demonstration of an end-to-end partially coherent deep optical neural network (PDONN) implemented on a silicon photonic chip, as shown in a simplified scheme in Fig. 1c^24^. Their architecture directly addresses two challenges in optical neural networks: limited network depth, due to insufficiently cascadable nonlinear activation functions, and restricted input dimensions, imposed by the requirements of coherent light sources and detection schemes. The PDONN introduces a monolithically integrated opto-electro-opto (O-E-O) nonlinear activation function based on micro-ring modulators driven by differential photocurrents. This configuration achieves positive net optical gain, enabling multiple nonlinear layers to be cascaded on a single chip without the need for external electrical amplifiers—a crucial step for realizing deeper photonic neural architectures. The network also integrates two convolutional layers and two fully connected layers in a compact 17 mm² silicon photonic chip with a 64-channel optical input, reporting the largest demonstrated input size and layer count for an end-to-end on-chip photonic neural network to date.

By operating with partially coherent optical sources such as amplified spontaneous emission (ASE) or LED-based emitters, the system avoids reliance on narrow-linewidth lasers and complex coherent detection, thereby enhancing manufacturability and robustness while maintaining high classification accuracy. The PDONN achieved 96% accuracy for binary fashion image classification and 94% for four-class handwritten digit recognition, with performance close to that obtained under fully coherent illumination. The design further benefits from real-valued optical computation, reducing the hardware complexity associated with phase control and improving tolerance to fabrication variations. The total latency per inference is approximately 4.1 ns, dominated by the RC delay of the O-E-O nonlinearities, and the estimated energy efficiency is ~122 pJ per operation. Together, these results demonstrate an optical computing platform that combines high speed, energy efficiency, and architectural scalability.

The work by Wu et al. exemplifies a broader shift in optical neural network research — from demonstrating individual components to realizing fully integrated computational pipelines. Their system showcases a self-contained optical neural processor capable of performing end-to-end inference at high speed, demonstrating both completeness and functional viability. Although such systems are still far from matching electronic accelerators in terms of scale or configurability, their architectural integrity makes them valuable platforms for testing and refining optical computing concepts under realistic conditions^25,26^. Continued co-optimization and experimental validation could pave the way for high-speed, application-specific systems, highlighting the potential of optical approaches to outperform electronic hardware in tasks requiring ultrafast inference.
